# Supplementary material for: Negative Photoconductivity in 2D α-MoO3/Ir Self-Powered Photodetector: Impact of Post-Annealing
Source: Materials (Basel). 2023 Oct 19;16(20):6756. doi: 10.3390/ma16206756 (PMC10608330; doi:10.3390/ma16206756)
Supplement: Supplementary file 1 [file materials-16-06756-s001.zip › materials-2645808-supplementary.pdf]

Additional Information

Article

Negative Photoconductivity in 2D α-MoO_3/Ir Self-Powered Photodetector: Impact of Post-Annealing

Mohamed A. Basyooni - M. Kabatas[1,2*], Shrouk E. Zaki[2], Khalid Rahmani[3], Redouane En-nadir[4] and Yasin Ramazan Eker[5,6]

[1] Department of Precision and Microsystems Engineering, Delft University of Technology, Mekelweg 2, 2628 CD Delft, Netherlands
[2] Department of Nanotechnology and Advanced Materials, Graduate School of Applied and Natural Science, Selçuk University, Konya 42030, Turkey
[3] Department of Physics, Ecole Normale Supérieure (ENS), Mohammed V University, Rabat, Morocco
[4] Laboratory of Solid-State Physics, Faculty of Sciences Dhar el Mahraz, University Sidi Mohammed Ben Abdellah, PO Box 1796 Atlas Fez 30 000, Morocco
[5] Department of Basic Sciences, Faculty of Engineering, Necmettin Erbakan University, Konya 42090, Turkey
[6] Science and Technology Research and Application Center (BITAM), Necmettin Erbakan University, 42090, Konya, Turkey

* Correspondence: Correspondence may be sent to Mohamed A. Basyooni - M. Kabatas (m.kabatas@tudelft.nl, m.a.basyooni@gmail.com)

Energy-dispersive X-ray spectroscopy (EDS):

Energy-dispersive X-ray spectroscopy (EDS) analysis is a valuable tool for examining the elemental composition of thin films. In the analysis of the Si substrate used in this study, as depicted in Figure 1, it is evident that silicon constitutes nearly 99.1% of the total weight, overshadowing the oxygen content. Consequently, the Si peak has been excluded from the results. Furthermore, the minimal oxygen concentration observed does not raise concerns, as it is unlikely to significantly affect the oxygen content of the MoO_3 film. Nonetheless, it remains intriguing to explore both scenarios—with and without silicon—and observe how the concentrations of all elements vary in these conditions.

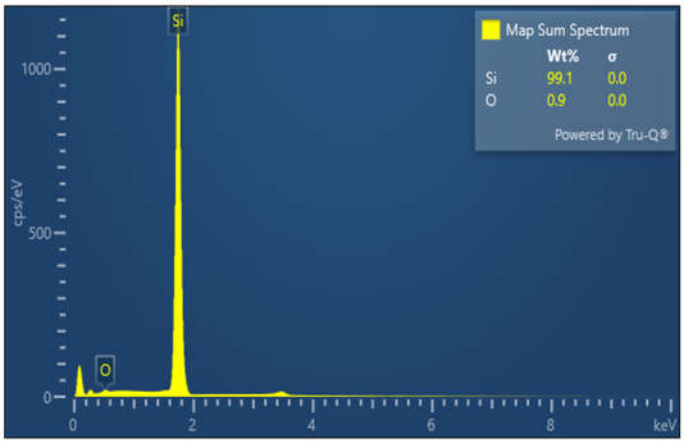

Figure S1 shows the EDX mapping of a pure n-type silicon substrate used in this study.

Table 1 presents the EDX findings for unannealed Mo/2 and 4 nm Ir/Si specimens. Mo content becomes apparent with readings of 0.4% and 0.7%, followed by Ir at 2.1% and 5.6%, and lastly, oxygen at 1.2% and 1.7%. EDS exhibits superior metal detection capabilities, enabling the precise identification of Ir concentrations that correspond to thickness variations. The minor fluctuations in Mo and O concentrations may be attributed to the presence of small particles with minimal deficiencies, a common expectation in EDS analysis. The analysis of EDS data involves the application of statistical techniques for data processing and interpretation. One such method, background subtraction, is employed in EDS analysis. EDS spectra encompass both signal and background noise, necessitating the utilization of statistical techniques to estimate and eliminate background noise, thereby enhancing analysis precision. Another statistical approach used in EDS analysis is peak fitting [8]. EDS spectra feature peaks that correspond to the elemental composition of the sample. Peak fitting entails the application of statistical methods to fit mathematical models to these spectral peaks, facilitating the determination of the elemental composition and quantity of the sample [9]. Consequently, it is anticipated that the detected signals will reveal small fractions. Interestingly, by excluding the Si peak, more suitable concentrations of Mo and Ir are identified. Specifically, Mo concentrations of 6.5% and 3.0% correspond to Ir concentrations of 64.5% and 78.0%, respectively. This outcome aligns with expectations, as the increased thickness of the Ir layer

leads to higher Ir concentrations and, in turn, decreased Mo concentrations in a similar fashion. Likewise, oxygen concentrations register at 29.0% and 18.9%.

The results for post-annealed samples are shown in Table 1. In the presence of silicon, the Mo concentration remains relatively constant at 0.1% and 0.2%. Conversely, the Ir content increases with Ir thickness, measuring 1.4% and 5.4%. Oxygen contents also exhibit a slight rise, reaching 1.7% and 2.7% as Ir thickness increases from 2 to 4 nm. These observations suggest a proportional increase in Ir content with greater Ir thickness. It is worth noting that post-annealing in an air environment leads to an increase in oxygen content. From a statistical perspective, adjusting the silicon and oxygen percentages to make up 100% can address potential discrepancies, reducing the need to focus extensively on silicon concentrations. Upon comparing these results to the unannealed samples, it becomes apparent that Mo content decreases, Ir content decreases, oxygen content increases, and silicon content decreases. These variations, both increases and decreases, are minor but expected due to the high-temperature annealing process, which can alter material concentrations within the samples. In the absence of the silicon peak, Mo concentrations are found to be 0.9% and 0%, while Ir concentrations are 56.1% and 72.6% for the 2 and 4 nm Ir thickness samples, respectively. Correspondingly, oxygen concentrations are 43.0% and 27.4%. These results reflect decreased Mo and oxygen content and an increased Ir content, consistent with the fact that metals like Ir tend to exhibit stronger signals in comparison to oxides and gases during detection.

Table S1 shows the elemental composition amounts of the prepared samples. The concentrations of all elements are recorded in Wt%.

	**Presence of Si**				**Absence of Si**		
**Samples**	**Si**	**O**	**Ir**	**Mo**	**O**	**Ir**	**Mo**
Unannealed Mo/2 nm Ir/SiO_2/Si	96.3	1.2	2.1	0.4	29.0	64.5	6.5
Unannealed Mo/4 nm Ir/SiO_2/Si	92.1	1.7	5.6	0.7	18.9	78.0	3.0
Post annealed Mo/2 nm Ir/SiO_2/Si	96.8	1.7	1.4	0.1	43.0	56.1	0.9
Post annealed Mo/4 nm Ir/SiO_2/Si	91.6	2.7	5.4	0.2	27.4	72.6	0.0

To gain a comprehensive understanding of element distribution within all the samples across the thin film, EDS layered mapping scans were conducted and generated elemental mapping distributions for the unannealed samples, as depicted in Figure 2. As anticipated, it observed well-distributed surface elements of Mo across all the samples with minimal interference and excellent integration. These findings underscore the intriguing potential of employing ALD for large-scale oxide production at nanometer scales. Furthermore, it's worth noting that all the samples exhibit homogeneity and repeatability.

In addition, the results for the post-annealed Mo/2 nm Ir/SiO_2/Si and Mo/4 nm Ir/SiO_2/Si samples are presented in Figure 3. In (a), some dust particles are visible, while in (b), we can observe the presence of particles. Nevertheless, in both cases, all the elements exhibit uniform distribution and homogeneity across the wafer sample.

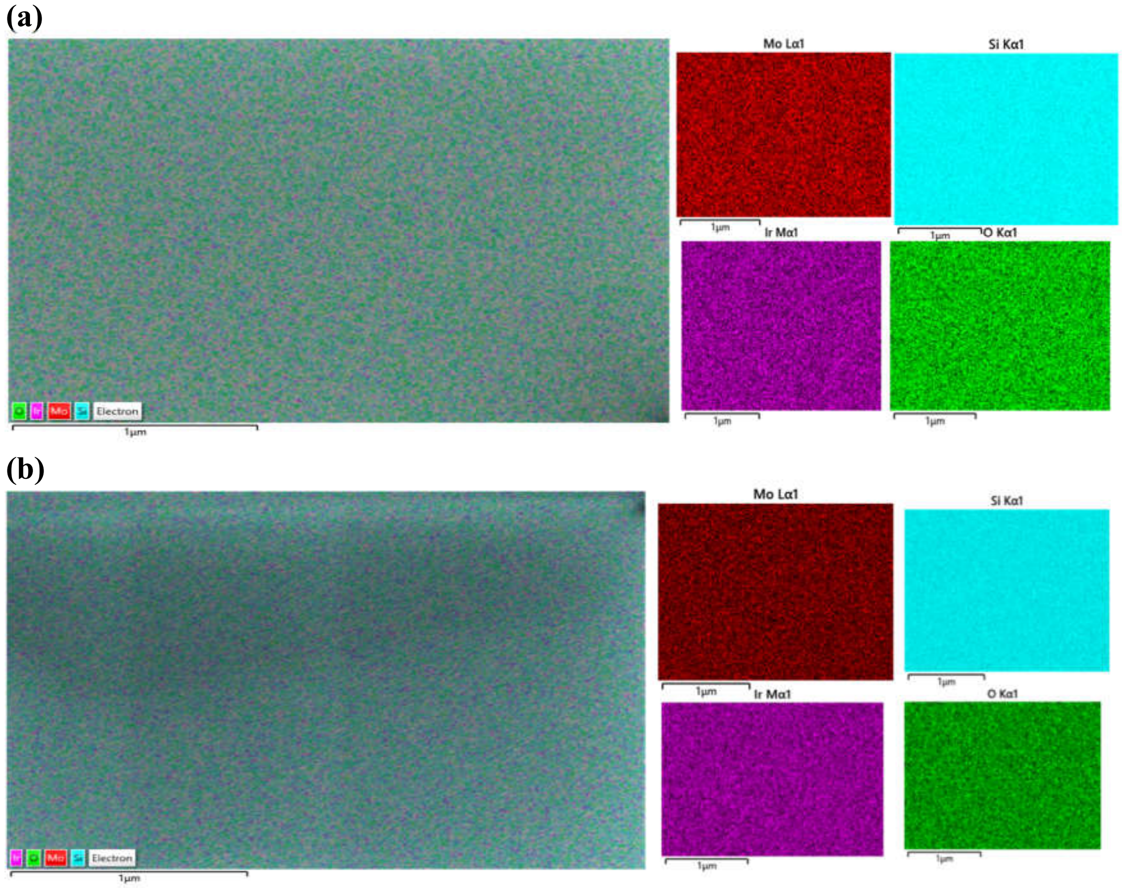

Figure S2. shows the Mo/Ir/SiO_2/Si EDX layered images and elemental mapping distributions of O, Si, Ir, and Mo elements of (a) Mo/2 nm Ir/SiO_2/Si, and (b) Mo/4 nm Ir/SiO_2/Si samples.

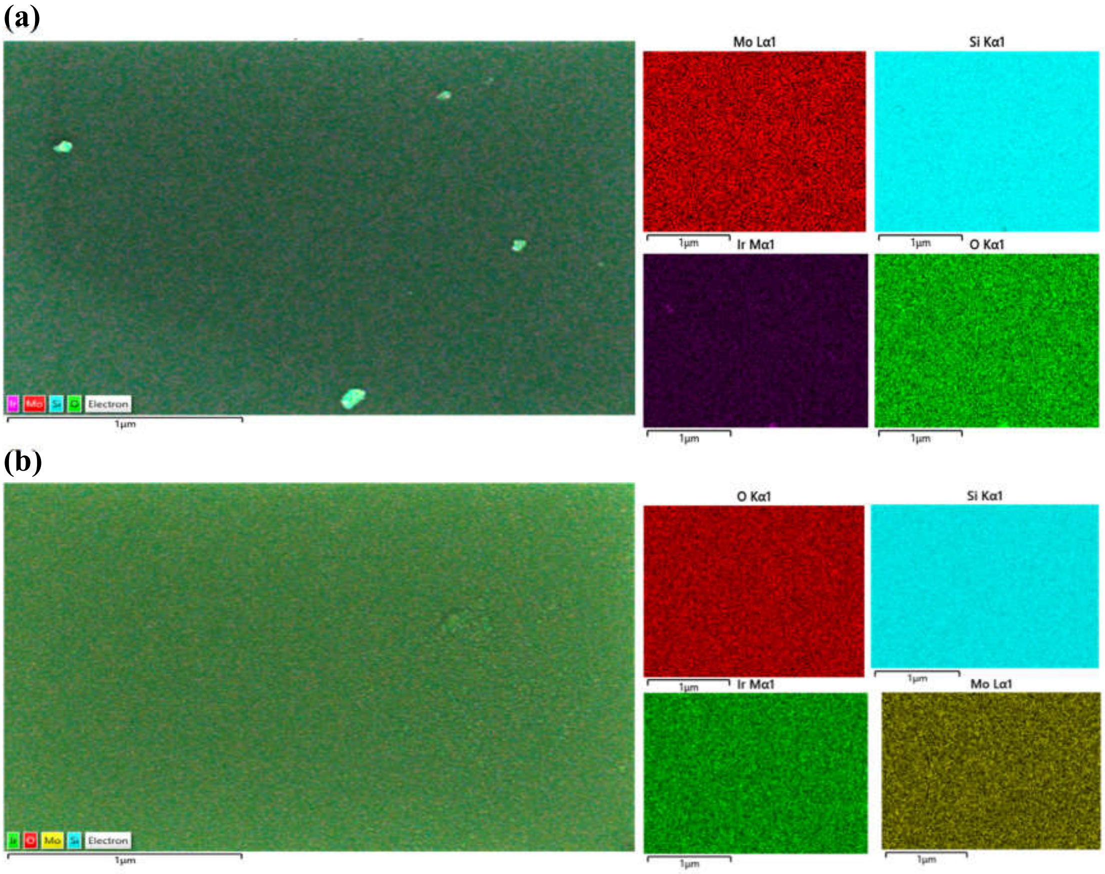

Figure S3. shows the EDS layered images and elemental mapping distributions of O, Si, Ir, and Mo elements of Mo/2 nm Ir/SiO_2/Si (a), and Mo/4 nm Ir/SiO_2/Si (b) samples.

References:

[1] J. Rath, A. Venkatesh, V. Moorthy, Ultra-Thin Plasmonic Optoelectronic Devices, Recent Advances in Thin Film Photovoltaics, Springer2022, pp. 219-271.
[2] H. Tang, C.-J. Chen, Z. Huang, J. Bright, G. Meng, R.-S. Liu, N. Wu, Plasmonic hot electrons for sensing, photodetection, and solar energy applications: A perspective, The Journal of Chemical Physics, 152 (2020) 220901.
[3] X. Chang, Y.-F. Wang, X. Zhang, R. Wang, Z. Liu, J. Fu, D. Zhao, F. Li, J. Wang, W. Wang, Iridium size effects in localized surface plasmon-enhanced diamond UV photodetectors, Applied Surface Science, 487 (2019) 674-677.
[4] J. Liu, F. Liu, H. Bai, W. Zhuang, Y. Xu, Effect of iridium doping on electronic structure and optical properties of m-BiVO4 photocatalytic materials: a first principles study, Molecular Physics, 120 (2022) e2002958.
[5] J. Weaver, C. Olson, D.W. Lynch, Optical investigation of the electronic structure of bulk Rh and Ir, Physical Review B, 15 (1977) 4115.
[6] Y.-F. Wang, X. Chang, S. Li, D. Zhao, G. Shao, T. Zhu, J. Fu, P. Zhang, X. Chen, F. Li, Ohmic contact between iridium film and hydrogen-terminated single crystal diamond, Scientific Reports, 7 (2017) 12157.
[7] S.F. Hung, F.X. Xiao, Y.Y. Hsu, N.T. Suen, H.B. Yang, H.M. Chen, B. Liu, Iridium Oxide-Assisted Plasmon-Induced Hot Carriers: Improvement on Kinetics and Thermodynamics of Hot Carriers, Advanced Energy Materials, 6 (2016) 1501339.
[8] J.I. Goldstein, D.E. Newbury, J.R. Michael, N.W. Ritchie, J.H.J. Scott, D.C. Joy, Scanning electron microscopy and X-ray microanalysis, Springer2017.
[9] Z. Chen, A.J. D'Alfonso, M. Weyland, D.J. Taplin, S.D. Findlay, L.J. Allen, Absolute-Scale Quantitative Energy Dispersive X-ray Analysis in Aberration-Corrected Scanning Transmission Electron Microscopy, Microscopy and Microanalysis, 21 (2015) 1079-1080.
[10] H. Xu, M.K. Akbari, Z. Hai, Z. Wei, L. Hyde, F. Verpoort, C. Xue, S. Zhuiykov, Ultra-thin MoO3 film goes wafer-scaled nano-architectonics by atomic layer deposition, Materials & Design, 149 (2018) 135-144.
[11] M. Dieterle, G. Weinberg, G. Mestl, Raman spectroscopy of molybdenum oxides Part I. Structural characterization of oxygen defects in MoO 3− x by DR UV/VIS, Raman spectroscopy and X-ray diffraction, Physical Chemistry Chemical Physics, 4 (2002) 812-821.
[12] L. Seguin, M. Figlarz, R. Cavagnat, J.-C. Lassègues, Infrared and Raman spectra of MoO3 molybdenum trioxides and MoO3· xH2O molybdenum trioxide hydrates, Spectrochimica Acta Part A: Molecular and Biomolecular Spectroscopy, 51 (1995) 1323-1344.
